# Supplementary material for: Effect of EGCG Extracted from Green Tea against Largemouth Bass Virus Infection
Source: Viruses. 2023 Jan 3;15(1):151. doi: 10.3390/v15010151 (PMC9864265; doi:10.3390/v15010151)
Supplement: Supplementary file 1 [file viruses-15-00151-s001.zip › viruses-2141008-supplementary.pdf]

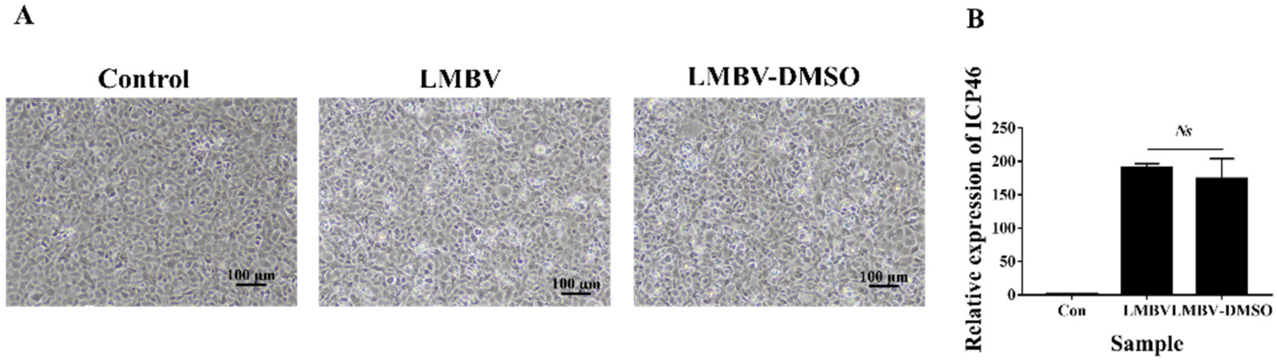

Supplementary Figure S1. The effect of DMSO on LMBV infection. FHM cells (1.5 × 106) were seeded in 12-well plates and incubated at 28 °C for 18 h. DMSO was diluted 8.33 times, which was same as diluted EGCG. Diluted DMSO was premixed with LMBV (MOI=1) as the test group. FHM cells incubated with LMBV (MOI=1) served as control group. FHM cells incubated with L15 medium alone served as blank group. (**A**) the CPEs were observed by light microscopy after 48h infection at 28 °C; (**B**) FHM cells and culture medium in each wells were used to extract total RNA, and the expression of the ICP46 gene was assessed by RT-qPCR at 48hpi. All the results showed that the addition of DMSO did not show an effect on LMBV infected FHM cells.

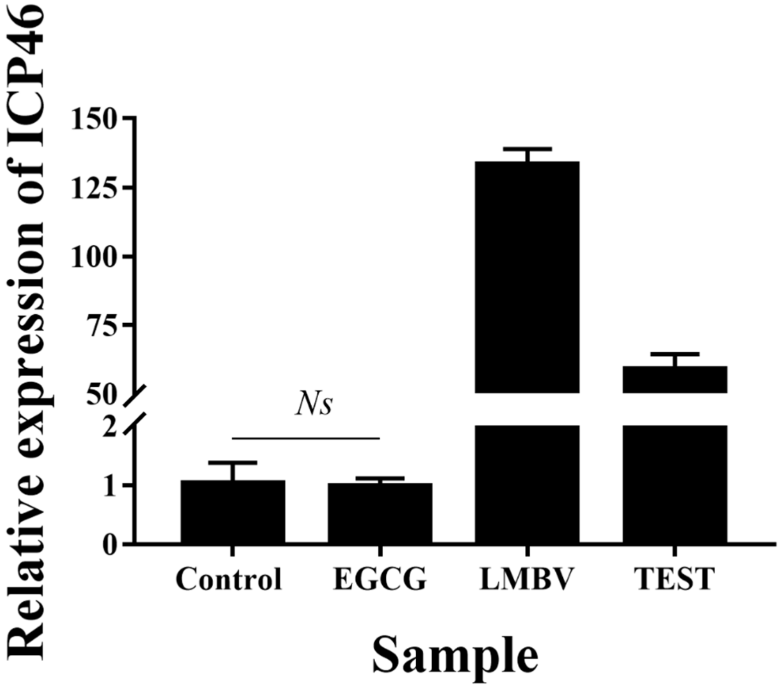

Supplementary Figure S2. The effect of EGCG on LMBV infection. Control was FHM cells with serum-free L15 medium. EGCG was FHM cells with EGCG at safe concentrations. LMBV was FHM cells with LMBV (MOI-1). TEST was FHM cells with EGCG at safe concentrations and LMBV (MOI-1).
